# EHMTI-0293. Comparison between celecoxib and prednisolone in bridge stage therapy following medication overuse headache: double blind randomized clinical trial

**DOI:** 10.1186/1129-2377-15-S1-C54

**Published:** 2014-09-18

**Authors:** M Togha, SMH Paknejad, E Taghvaei, T Ramim, S Razeghi jahromi

**Affiliations:** 1 Iranian Center of Neurological Research-Neuroscience Institute, Tehran University of Medical Sciences, Tehran, Iran; 2 Multiple Sclerosis Research Center-Neuroscience Institute, Tehran University of Medical Sciences, Tehran, Iran

## Introduction

Many patients with chronic or frequent headaches overuse the analgestic or anti-migraine drugs. Medication overuse headache (MOH) is a common severe headache. The basis of treatment of MOH is discontinuation of the offending drugs. However, cessation of drug consumption could lead to severe disabling headache. Corticosteroids are the most popular drugs for bridge phase of treatment. However corticosteroids might cause different side effects. Celecoxib is a Cox 2 inhibitor drug with analgesic and anti-inflammatory properties, assumed to be a good alternative for steroids.

## Aims

We conducted this study to compare celecoxib with prednisolone for bridge stage therapy in MOH.

## Methods

103 patients (18-65 years) with at least 15 days of headache per month were enrolled. They assigned into two groups: celecoxib (53 cases) and prednisolone (51 cases). Prednisone was administered as daily doses of 75, 50, 30, 25, and 10 mg in 3 days interval. Celecoxib was administered for 15 days according to the following schedule: 100 mg three times per day (5 days), twice a day (5 days) and once a day (5 days). The severity and duration of headache, as well as side effects were assessed.

## Results

No significant differences were observed in the decrement of the severity and duration (p=0.114 and 0.149 respectively) of headache between two groups. Side effects were reported in 42 % and 18.9% of prednisolone and celcoxibe groups respectively (Relative Risk=2.2, p=0.011).

## Conclusion

Celecoxib has similar efficacy and fewer side compared to prednisolone during withdrawal therapy.

No conflict of interest.

